# Perforated Jejunal Diverticulum Mimicking Diverticulitis: A Case Report of Acute Abdominal Pain in an Elderly Patient

**DOI:** 10.7759/cureus.68935

**Published:** 2024-09-08

**Authors:** Esteban Tapias, Eliesther F Rivera, Brooke Finlayson, Danielle A Rowe, Heather L Mateja, Landry K Umbu, Peter M DeVito

**Affiliations:** 1 General Surgery, Western Reserve Health Education/NEOMED (Northeast Ohio Medical University), Warren, USA; 2 Surgery, American University of Antigua, Osbourn, ATG; 3 Surgery, Ross University School of Medicine, Bridgetown, BRB; 4 College of Medicine, American University of Antigua, Osbourn, ATG; 5 General Surgery, American University of Antigua, Osbourn, ATG

**Keywords:** abdominal pain, acute abdomen, diverticulitis, jejunal diverticulitis, perforation

## Abstract

Jejunal diverticulosis is a rare form of diverticulosis characterized by acquired pseudodiverticula in the small bowel. Although most cases are asymptomatic, the condition can present diagnostic challenges due to its atypical presentation. Complications such as perforation can lead to acute abdomen, significantly increasing morbidity and mortality. We report a rare case of perforated jejunal diverticulitis in an 84-year-old female with a known history of diverticular disease. The patient presented to the emergency department with worsening left lower quadrant abdominal pain. A computed tomography (CT) scan revealed a focus of air adjacent to the mesentery, indicative of bowel perforation. An urgent exploratory laparotomy was performed, which identified a 4-cm perforated jejunal diverticulum. The affected segment of the small bowel was resected, followed by primary side-to-side jejunal anastomosis. The patient was discharged home in stable condition following uneventful postoperative recovery.

This case highlights the importance of including jejunal diverticulosis in the differential diagnosis of acute abdomen, particularly in elderly patients with a history of diverticular disease. Due to the nonspecific presentation, prompt imaging is crucial for diagnosis. Surgical intervention is often necessary in cases of perforation. Increased clinical awareness of this rare condition may help reduce diagnostic delays and improve patient outcomes.

## Introduction

Jejunal diverticulosis is a rare variant of diverticulosis that occurs within the gastrointestinal tract. The pathogenesis of jejunal diverticula, similar to colonic diverticula, involves the herniation of the mucosal and submucosal layers through the muscular wall [1,2]. Diverticula are most commonly found in the sigmoid colon, affecting approximately half of the elderly population in Western societies [3-6]. Although less common, diverticula can also develop in the small bowel, particularly in the jejunum, and to a lesser extent, in the ileum [7]. In contrast to colonic diverticulosis, jejunal diverticulosis presents significant diagnostic challenges due to its rarity and nonspecific symptoms. It is typically asymptomatic and often discovered incidentally during imaging or surgery performed for unrelated conditions [1,8,9]. However, jejunal and ileal diverticula are more prone to complications, including diverticulitis, bleeding, obstruction, or perforation, which can occur in up to 10% of affected patients [4]. It is more commonly diagnosed in males during their sixth to seventh decade of life [1,2].

This case report presents an instance of acute perforated jejunal diverticulitis in an 84-year-old female patient. This report's significance lies in the rarity of the condition and in highlighting the increasing need for clinical awareness, especially as the incidence of diverticular disease continues to increase [10]. We aim to raise awareness of this uncommon condition, with the goal of facilitating swifter diagnosis and management.

## Case presentation

An 84-year-old female with a past medical history of hypertension, gastroesophageal reflux disease, diverticulitis, hip osteoarthritis, and chronic back pain with an implanted spinal cord stimulator presented to the emergency department with worsening left lower quadrant abdominal pain for two days, accompanied by nausea and decreased appetite. She reported similar symptoms during previous diverticulitis episodes. She denied fever, chills, diarrhea, urinary symptoms, chest pain, or difficulty breathing. Her last bowel movement was the previous day.

**Figure 1:**
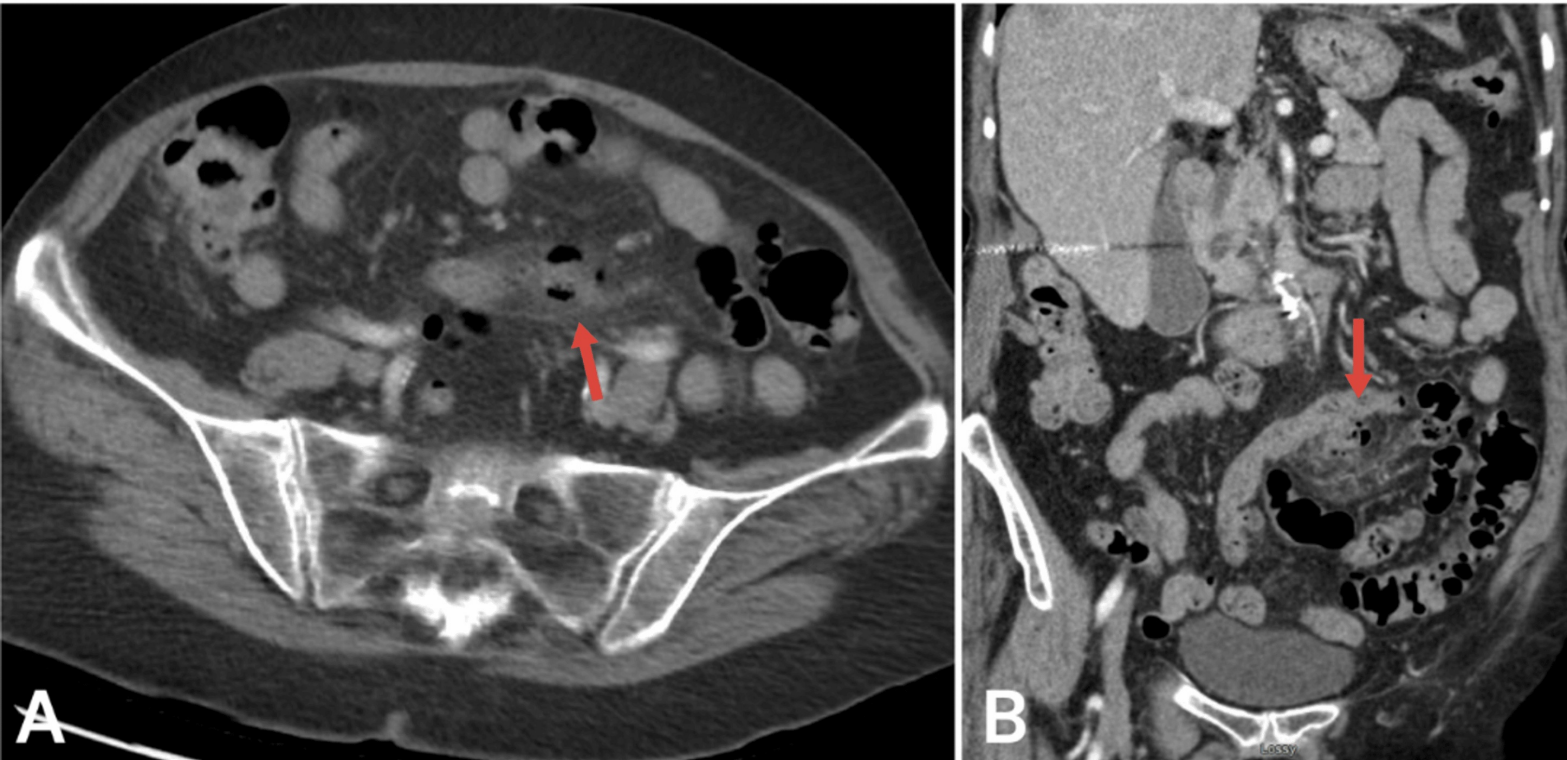
Abdomen and pelvis CT with intravenous IV contrast (a) Axial view of small bowel perforation and mesenteric air, (b) Coronal view of a small bowel diverticular perforation with significant fat stranding

On assessment, her vital signs were within normal limits. She appeared nontoxic, in no acute distress, and was afebrile. Abdominal examination revealed a soft abdomen with mild left lower quadrant tenderness and no signs of peritonitis. Laboratory results were unremarkable except for a lactate of 2.6 mmol/L and a C-reactive protein of 38 mm/hr. Intravenous (IV) fluids, antiemetics, and pain management were initiated. A computed tomography (CT) scan with IV contrast revealed a thickened loop of small bowel with inflammatory changes and a focus of air in the adjacent mesentery, suggesting focal perforation (Figure 1). The patient was started on ceftriaxone and metronidazole while awaiting surgery.

Overnight, her abdominal symptoms worsened, with increased nausea and left lower quadrant rebound tenderness, though she remained soft without involuntary guarding. Given the CT findings and clinical deterioration, she was taken for an exploratory laparotomy. Intraoperatively, purulent exudative fluid was identified, and a perforated jejunal diverticulum 60 cm distal from the ligament of Treitz was discovered. A 13 cm segment of the small bowel was resected, and a side-to-side isoperistaltic jejunal anastomosis was created with a stapler. The anastomosis was imbricated with 3-0 Vicryl and a crotch suture. The bowel was reinspected from the ligament of Treitz to the cecal valve, and no other perforations were found. The specimen was sent to pathology. 

**Figure 2:**
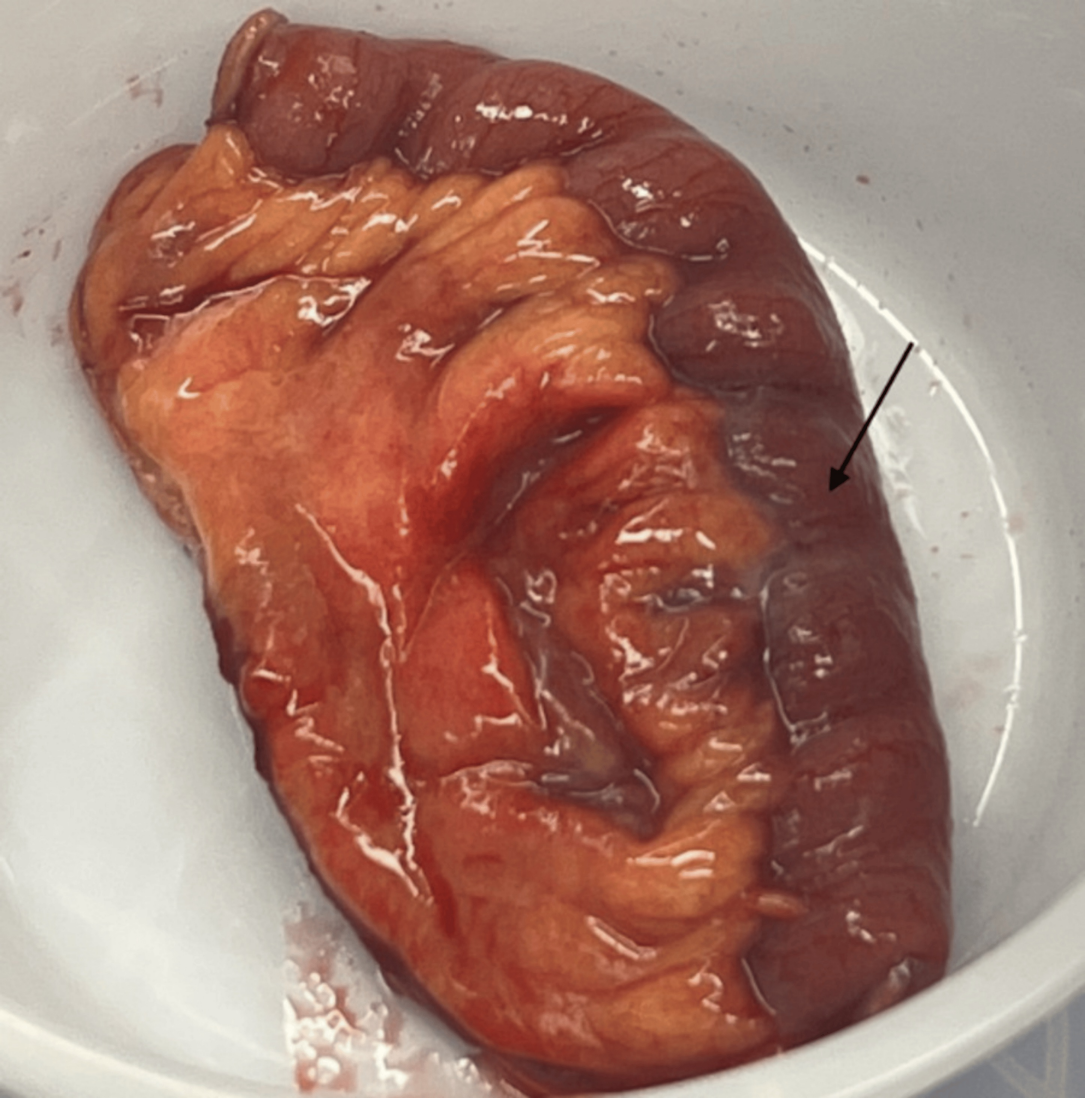
Gross surgical specimen A 13 cm segment of jejunum with a perforated diverticulum (black arrow) leading to inflamed, indurated, hemorrhagic adipose tissue.

Pathology revealed erythematous and congested bowel serosa, with a perforated diverticulum 4 cm from the resection margin leading to inflamed, indurated, hemorrhagic adipose tissue (Figure 2). A small abscess was noted within the mesenteric parenchyma. Cultures from the abdominal fluid were negative. The patient was monitored in the intensive care unit (ICU) for two days, during which she experienced repeated nausea without vomiting, consistent with her baseline symptoms, managed with ondansetron. She recovered well, with bowel function returning on post-op day four, and her diet was appropriately advanced. Antibiotics were discontinued on postop day five, and she was discharged without complications, with a follow-up scheduled in two weeks.

## Discussion

Diverticular disease is a common diagnosis affecting various gastrointestinal tract (GIT) segments, although it is primarily diagnosed in the colon [7,9]. Small bowel diverticulum, which can be congenital, as seen with Meckel’s diverticulum, or acquired, is a less frequently seen condition [11]. When present, nearly 80% of reported cases occur in the proximal jejunum while 15% of cases are found in the ileum [7]. Additionally, up to 5% of small bowel diverticula simultaneously involves the jejunum and ileum [7]. Jejunum diverticulum, a rare type of acquired pseudodiverticulum, was first described in 1794 by Somerling, who reported a case of diverticulum involving the jejunum and ileum [1,8]. The condition has an annual incidence of 0.6-1.3% [2,9]. Its prevalence is approximately 0.5-2.3% in contrast-enhanced imaging studies and 1.3-2.3% in post-mortem evaluation [1,7,8]. The majority of cases are seen in patients during their sixth to seventh decade of life, with a predilection for male patients [1,2,8,9]. Furthermore, there is evidence for a familial tendency and an association with systemic connective tissue disorders [9].

The etiology behind these diverticula is not entirely established [1]. However, it may be attributed to disruption in neuromotor innervation, leading to defective peristalsis, gut dyskinesia, and risk factors causing elevated intraluminal pressure [1,2,9]. Like colonic diverticulosis, the increased pressure contributes to the formation of false diverticula at the mesenteric border, particularly at weak points of the bowel wall where the vasa recta penetrates it [1,2,7,9,12]. Only the mucosal and submucosal layers are involved in this process, as opposed to muscular layer involvement noted in true diverticula [1,2]. To further differentiate, Meckel’s diverticulum is a type of true congenital diverticula present on the antimesenteric side of the bowel [13]. The size of these jejunal diverticula varies significantly, ranging from just a few millimeters to measuring above 10 centimeters [12].

Uncomplicated cases are mostly asymptomatic; however, when symptoms are present, these can be very unspecific [1,8,9]. The clinical presentation may resemble other causes of acute abdomen, making it challenging to reach a diagnosis [1,8,9]. Some symptoms experienced include abdominal pain, nausea, vomiting, and malabsorption [2,7,8,9]. Approximately 30-40% of cases present acutely with gastrointestinal bleeding, bowel obstruction, strangulation, volvulus, and peritonitis [1,2,7-9]. Although rare, jejunum diverticulum can be complicated by bowel perforation, occurring in only 2.3-6.4% of cases [2,8]. Initial evaluation should include evaluation for risk factors such as recent abdominal procedures, history of cancer, inflammatory bowel disease, diverticular disease, and foreign body ingestion [2]. Additionally, patients should be assessed for findings indicative of focal or diffuse peritonitis during physical examination [2]. Given their mostly asymptomatic nature, jejunal diverticulosis is often diagnosed incidentally during imaging studies or surgery for a different condition [8,9].

Diagnostic computed tomography (CT) is the preferred imaging modality since it identifies the presence, site, and possibly the cause of perforation, as well as signs of other possible complications [7,13]. In cases of perforated jejunal diverticulosis, CT findings may demonstrate free intra-peritoneal air, air adjacent to the bowel wall, asymmetric bowel wall thickening, edema, and fat stranding [13]. While abdominal X-ray is often employed in the initial assessment of acute abdomen, it rarely contributes to diagnosing jejunal diverticulosis [13]. Although these modalities aid in diagnosis, they can sometimes miss the presence of diverticulum, and some cases eventually necessitate surgical intervention via laparoscopy or exploratory laparoscopy for definite diagnosis [7]. The differential diagnosis in cases of jejunal diverticula perforation should include Crohn’s disease, small bowel malignancy, and trauma by a foreign body [1].

In uncomplicated cases, conservative management with intravenous fluids, bowel rest, and antibiotics can successfully treat the symptoms [2,9]. Peritonitis following jejunal perforation may be self-limited in localized cases [8]. However, in patients presenting with diffuse peritonitis, perforation, and hemodynamic instability, prompt resuscitation, and surgical evaluation are essential [2,9]. The preferred operative approach is open segmental resection of involved intestinal segments with primary anastomosis [7-9]. Careful examination of the entire bowel is essential to identify concurrent disease missed during imaging [2]. Furthermore, the excision of a single diverticulum carries a higher risk of sepsis, postoperative leak, and death [1]. Cases in which diagnostic laparoscopy was initially done may eventually be converted into an exploratory laparotomy [2,9]. Jejunal diverticula have a mortality rate of 0-5%, increasing to 40% in cases complicated by perforation, especially in patients with comorbidities, delayed diagnosis, and inappropriate management [2,7,8]. The mortality rate can decrease to 14% in cases managed by segmental bowel resection [8]. Mortality is higher when other surgical interventions are implemented such as simple closure, excision, and invagination [8].

## Conclusions

Jejunal diverticulitis presents significant diagnostic and therapeutic challenges due to its rarity. The difficulty in diagnosing and the absence of distinct symptoms further complicate its management. The causes of jejunal diverticulosis are not well understood; some mechanisms for the formation of diverticula include loss of colonic innervation or adverse side effects of certain medications. Perforation of the jejunal diverticulum carries an increased risk for mortality and should be included in the differential diagnosis for acute abdomen, particularly in elderly patients with a history of gastrointestinal conditions. Early diagnosis and urgent surgical intervention are crucial in preventing mortality in cases of jejunal diverticulitis with perforation. This case highlights the importance of these strategies in managing this rare yet potentially life-threatening condition.
